# Loss of RASSF2 Enhances Tumorigencity of Lung Cancer Cells and Confers Resistance to Chemotherapy

**DOI:** 10.1155/2012/705948

**Published:** 2012-05-24

**Authors:** Jennifer Clark, Jessica Freeman, Howard Donninger

**Affiliations:** Molecular Targets Program, Department of Medicine, James Graham Brown Cancer Center, University of Louisville, 505 S. Hancock Street, Louisville, KY 40202, USA

## Abstract

RASSF2 is a novel pro-apoptotic effector of K-Ras that is frequently inactivated in a variety of primary tumors by promoter methylation. Inactivation of RASSF2 enhances K-Ras-mediated transformation and overexpression of RASSF2 suppresses tumor cell growth. In this study, we confirm that RASSF2 and K-Ras form an endogenous complex, validating that RASSF2 is a bona fide K-Ras effector. We adopted an RNAi approach to determine the effects of inactivation of RASSF2 on the transformed phenotype of lung cancer cells containing an oncogenic K-Ras. Loss of RASSF2 expression resulted in a more aggressive phenotype that was characterized by enhanced cell proliferation and invasion, decreased cell adhesion, the ability to grow in an anchorage-independent manner and cell morphological changes. This enhanced transformed phenotype of the cells correlated with increased levels of activated AKT, indicating that RASSF2 can modulate Ras signaling pathways. Loss of RASSF2 expression also confers resistance to taxol and cisplatin, two frontline therapeutics for the treatment of lung cancer. Thus we have shown that inactivation of RASSF2, a process that occurs frequently in primary tumors, enhances the transforming potential of activated K-Ras and our data suggests that RASSF2 may be a novel candidate for epigenetic-based therapy in lung cancer.

## 1. Introduction

RASSF2 is a member of the RASSF family of proteins which consists of 10 family members (RASSF1–10). While all the family members are characterized by a conserved RalGDS/AF6 Ras association (RA) domain either in the C-terminal (RASSF1–6) or N-terminal of the protein (RASSF7–10), only RASSF1–6 contain a conserved SARAH (Salvador/RASSF/Hpo) domain adjacent to the RA domain [1–3]. It is well established that RASSF1–6 have tumor suppressor activity, and recent evidence suggests that other members of the family may also function as tumor suppressors [1, 3–8].

Although RASSF2 is structurally related to the better characterized RASSF1A, the mechanisms by which these two family members promote cell death may differ as RASSF2 localizes predominantly to the nucleus [9, 10] whereas RASSF1A is found primarily in the cytoplasm. RASSF2 binds to K-Ras in a GTP-dependent manner [11] and may serve as a K-Ras-specific effector as it forms an endogenous complex with K-Ras [12]. RASSF2 has no apparent intrinsic enzymatic activity or DNA binding properties and thus acts by interacting with other proapoptotic effectors and tumor suppressors, including PAR-4 [13] and the MST1/2 kinases [14, 15], thereby regulating the pathways these effectors control.

Like RASSF1A, RASSF2 is inactivated in a variety of tumors by promoter methylation [8, 9, 11, 13, 16–23]. RASSF2 has the properties of a tumor suppressor in that its overexpression promotes apoptosis and cell cycle arrest *in vitro * and inhibits tumor cell growth and tumor xenograft formation in nude mice [9, 11]. Conversely, loss of RASSF2 expression results in enhanced growth in soft agar and transformation [24]. Loss of RASSF2 may also promote metastasis [23, 25]. RASSF2 may function in additional biological processes other than apoptosis and growth suppression as suggested by *RASSF2* knockout mice. These mice develop normally for the first two weeks after birth, where after they develop growth retardation and die approximately 4 weeks after birth [26]. Additionally, these mice develop systemic lymphopenia and altered bone development. This suggests that RASSF2 has important functions in early post-natal development and further confirms that RASSF2 has functions distinct from RASSF1A as *RASSF1A* knockout mice develop normally [27, 28].

Although RASSF2 is expressed in a wide variety of tissues [26], its expression is somewhat tissue specific, with the highest levels detected in brain, peripheral blood, and lung [11]. RASSF2 is frequently downregulated in lung cancer [9, 11, 19] with inactivation of RASSF2 being more prevalent in NSCLC than SCLC. K-Ras is frequently mutated in lung cancer [29], and inactivation of RASSF2 enhances the transforming potential of K-Ras in rat kidney cells [24]. Several reports indicate that there is a positive correlation between K-Ras/BRAF mutations and *RASSF2* methylation in primary tumors [21, 24, 30]. Thus, inactivation of RASSF2 confers a growth advantage to tumor cells harboring activated K-Ras, and loss of RASSF2 expression may be a key event in Ras-mediated transformation.

To date, the majority of studies examining the effects of RASSF2 on the transformed phenotype rely on overexpression assays, which although providing useful information, have some drawbacks in that overexpression of proteins from viral promoters may yield expression levels far above physiological levels, thereby generating data that may not be physiologically relevant. We have used RNAi technology to reduce RASSF2 expression levels, a situation that more accurately mimics what occurs in primary tumors, to determine the role of RASSF2 inactivation in transformation. Loss of RASSF2 expression in lung cancer cells dramatically enhanced the transformed phenotype, decreased cell adhesion, and increased invasion. These effects were associated with elevated levels of activated AKT. Furthermore, inactivation of RASSF2 conferred resistance to taxol and cisplatin, suggesting that RASSF2 may be a target for epigenetic therapy in lung cancer.

## 2. Materials and Methods

### 2.1. Cell Lines and Culture Conditions

 H441 lung cancer cells were maintained in RPMI1640 (Mediatech Inc., Herndon, VA) supplemented with 10% fetal bovine serum (FBS; Mediatech Inc.) and 1% penicillin-streptomycin (Mediatech Inc.).

### 2.2. Knockdown of RASSF2 by Short Hairpin RNA

H441 cells were transfected with pLKO.1 lentiviral constructs encoding shRNA molecules to RASSF2 with the following sequences: shF2 number 1, 5′-TCTGAAGACCTACAACTTGTA-3′ and shF2 number 2, 5-GCCACCGATTACCCGCTGATT-3′, and a control shRNA that corresponded to RASSF2 sequences but which was ineffective at reducing RASSF2 levels 5′-CCTCCCAAGTAGCTGGAATTA-3′ (Open Biosystems, Lafayette, CO) using Lipofectamine 2000 (Invitrogen, Carlsbad, CA) and selected with puromycin to obtain a stable bulk population of cells.

### 2.3. Western Blot Analysis

Total cell lysates were prepared by lysing the cells in RIPA buffer (Sigma, St. Louis, MO) supplemented with 100 *μ*g/mL leupeptin, 100 *μ*g/mL aprotinin, and 1 mM sodium orthovanadate. The lysates were passed through a 21-gauge needle, centrifuged to remove debris, and quantitated using the BioRad Protein Assay (BioRad, Hercules, CA). Equal amounts of protein were resolved on 4–12% NuPage Novex polyacrylamide gels (Invitrogen) and incubated with antibodies against RASSF2 [11], *β*-Actin (Sigma), phospho-AKT (9271), and AKT (9272) (Cell Signaling Technology, Inc., Danvers, MA). The signal was detected by enhanced chemiluminescence.

### 2.4. Immunoprecipitation

Endogenous coimmunoprecipitations of Ras and RASSF2 were performed using a Pan-ras antibody conjugated to sepharose beads (Santa Cruz Biotechnology Inc., Santa Cruz, CA) to immunoprecipitate the lysate. The immunoprecipitates were then analyzed by Western Blot using our RASSF2 antibody [11].

### 2.5. Cell Proliferation Assays

2 × 10^5^ cells per well were plated in 6-well plates and incubated for 6 days. Cell number was determined each day by counting the number of viable cells in one well of each plate for the different cell lines. Experiments were performed twice in duplicate.

### 2.6. Cytotoxicity Assays

5 × 10^4^ cells per well were plated in 12-well plates and incubated with 5 nM taxol, 50 *μ*M cisplatin, or vehicle for 3 days. The number of surviving cells was determined by cell counting. Experiments were performed twice in duplicate.

### 2.7. Soft Agar Assays

1 × 10^4^ cells were plated in 6 mL of 0.35% agar in complete growth medium overlaid on a 0.7% agar base, also in complete growth medium. The cells were incubated at 37°C for 2 weeks and resulting colonies were counted after staining for 16 hr with p-iodonitrotetrazolium violet. Experiments were performed twice in duplicate.

### 2.8. Adhesion Assays

Cell adhesion assays were performed essentially as described [31]. Briefly, 5 × 10^4^ cells per well were plated in BSA-coated 96-well plates and allowed to adhere for 45 min at 37°C. The medium was removed and the adhering cells fixed and stained with crystal violet. The dye was solubilized, and absorbance at 570 nm was used as a measure of adhesion.

### 2.9. Invasion Assays

1 × 10^5^ cells per well were plated on a collagen plug in serum-free growth medium in transwell inserts. The inserts were placed in 12-well plates containing complete growth medium and incubated at 37°C for 7 days. Cells on the inner surface of the transwell membrane were removed by scraping with a cotton swab, and cells remaining on the outer surface of the membrane were fixed and stained with crystal violet. The number of cells remaining on the outer surface of the transwell membrane was then quantitated by cell counting.

## 3. Results

### 3.1. RASSF2 Forms an Endogenous Complex with K-Ras

**Figure 1:**
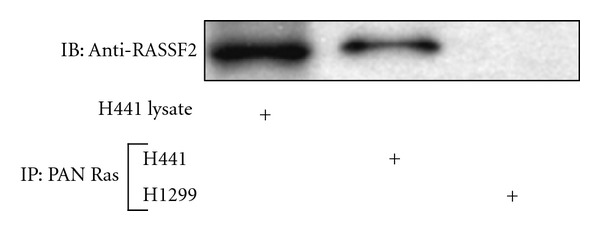
RASSF2 and K-Ras form an endogenous complex. Lysates from H441 and H1299 lung cancer cells were immunoprecipitated with a pan Ras antibody, fractionated on SDS gels, and immunoblotted with an anti-RASSF2 antibody. The endogenous interaction between Ras and RASSF2 was confirmed by the presence of RASSF2 in the proteins precipitated from the H441 cells but not the RASSF2-negative H1299 cells.

RASSF2 has previously been shown to directly bind to K-Ras *in vitro* in a GTP-dependent manner [11]. To confirm that RASSF2 and K-Ras can form an endogenous complex, we serum-starved then briefly serum-stimulated H441 lung cancer cells that express mutant K-Ras and retain RASSF2 expression [11]. The cells were then lysed and immunoprecipitated with a pan-Ras antibody conjugated to sepharose beads and the immunoprecipitate subjected to Western Blotting with a RASSF2 antibody [11] (Figure 1). The presence of RASSF2 in the immunoprecipitate confirmed that the interaction between RASSF2 and K-Ras is physiologically relevant and RASSF2 is a *bone fide* Ras effector.

### 3.2. Downregulation of RASSF2 Enhances the Proliferation of Tumor Cells

**Figure 2:**
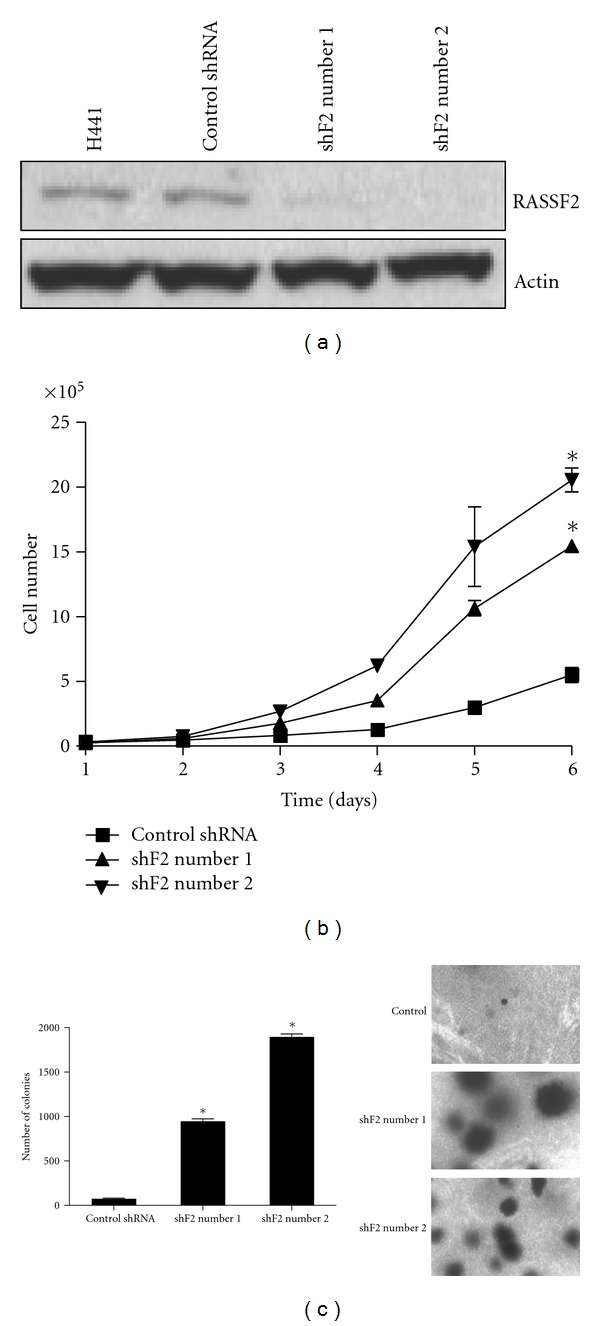
Loss of RASSF2 enhances proliferation and tumorigenicity of lung cancer cells. H441 lung cancer cells were transfected with two independent RASSF2 shRNA constructs and a noneffective shRNA and selected in puromycin for 2 weeks to obtain a population of cells stably expressing the various shRNA constructs. Knockdown of RASSF2 expression was confirmed by Western Blotting (a). Actin was used as a control for protein loading. (b) Growth analysis of the H441 shF2 cells. Cells were harvested and counted at the indicated times to determine cell number. *P* < 0.05 for both shF2-transfected cells compared to control cells. (c) H441 cells stably expressing the shRNA constructs to RASSF2 or control shRNA were plated in soft agar and colony number determined after 14 days. *Statistically different (*P* < 0.05) from cells expressing the control shRNA. The panel on the right shows representative images of the colonies.

To determine the biological effects of downregulating RASSF2, we used two independent RASSF2 shRNA constructs to generate stable RASSF2 knockdown cell lines in H441 lung cancer cells. An shRNA molecule that did not knockdown RASSF2 was used as a control. Knockdown of RASSF2 expression in the H441 cells was validated by Western Blotting using our RASSF2 antibody (Figure 2(a)). Analysis of cell proliferation confirmed that the RASSF2 knockdown cells exhibited statistically significant (*P* < 0.05) enhanced proliferation compared to control cells (Figure 2(b)).

### 3.3. Loss of RASSF2 Expression Promotes the Transformed Phenotype

To determine the effects of loss of RASSF2 expression on the transformed phenotype, we plated the H441 RASSF2 knockdown cells in soft agar and compared their ability to form colonies with that of the control cells (Figure 2(c)). The plates were examined 2 weeks after plating and scored for the number of colonies. The cells in which RASSF2 had been knocked down formed significantly more colonies than the control cells (*P* < 0.05) and the colonies that formed were also much larger (Figure 2(c)). These results are consistent with previous reports showing that inactivation of RASSF2 enhances K-Ras-induced cell transformation [24].

### 3.4. Inactivation of RASSF2 Results in a More Aggressive Phenotype

**Figure 3:**
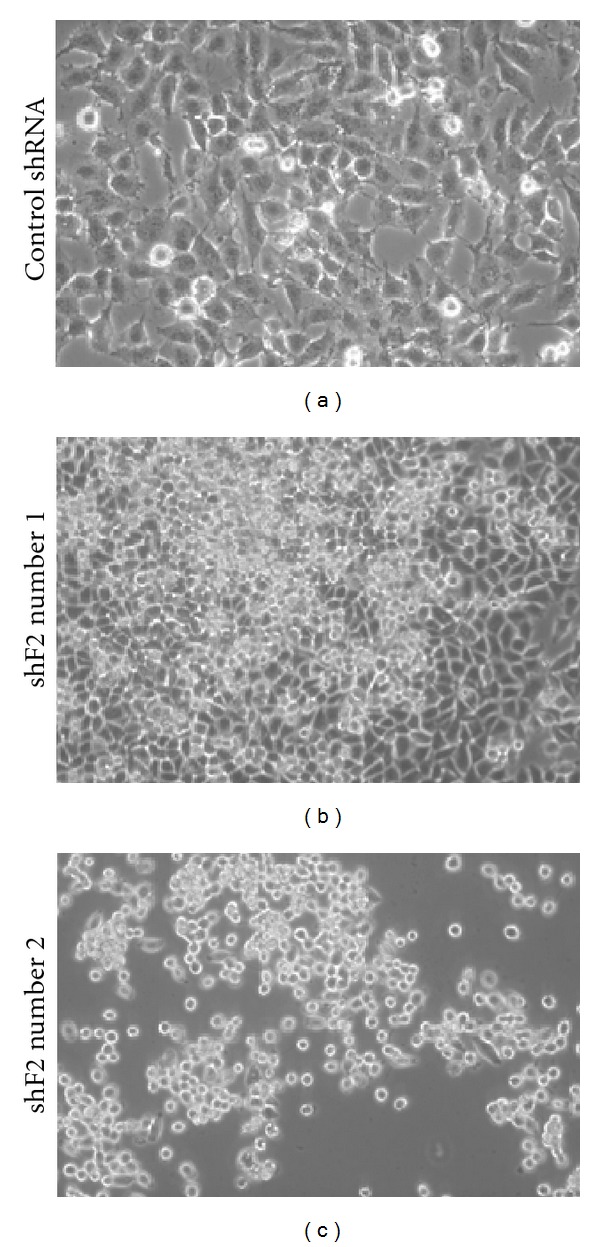
Inactivation of RASSF2 causes morphology changes. H441 cells stably expressing RASSF2 shRNA constructs and a control shRNA were viewed and photographed using phase contrast at 100x magnification.

Overexpression of RASSF2 has been shown to induce cell morphological changes [24], and we have confirmed this in our RASSF2 knockdown cells. Loss of RASSF2 expression resulted in a dramatic alteration in cell morphology. The control H441 cells had a flattened morphology and grew in a monolayer, whereas the cells stably expressing the RASSF2 shRNA constructs became more rounded, piled up on each other, and were more refractile, consistent with a more aggressive and transformed phenotype (Figure 3).

**Figure 4:**
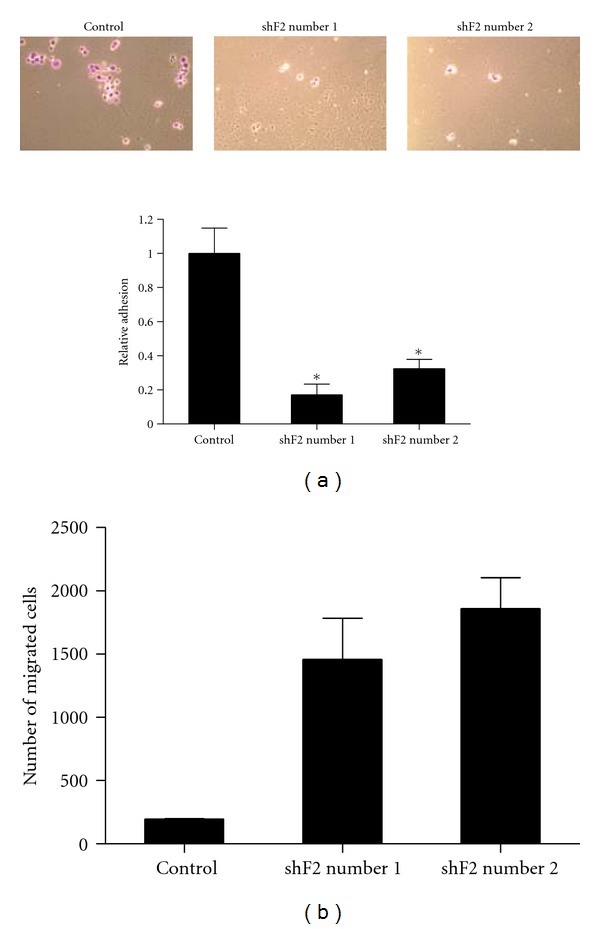
Loss of RASSF2 decreases cell adhesion and enhances invasion. (a) The H441 control cells and those stably knocked down for RASSF2 were assayed for adhesion as described in Section 2. *Statistically different (*P* < 0.05) from control cells. (b) The cells were assayed for their ability to invade a collagen matrix as described in Section 2. A statistically larger number of cells (*P* < 0.05) that were stably knocked down for RASSF2 were able to migrate through the collagen compared to control cells, indicating that loss of RASSF2 enhances cell invasion.

The RASSF2 knockdown cells also exhibited a significant decrease in the degree of adhesion compared to the control cells (Figure 4(a)), a characteristic frequently associated with transformed cells that correlates with enhanced motility. In addition, loss of RASSF2 expression enhanced invasion of the cells. Significantly more cells stably expressing the RASSF2 shRNA constructs were able to invade through a collagen matrix compared to control cells (Figure 4(b)). This result is in agreement with other published reports showing that over expression of RASSF2 inhibits migration [23]. Taken together, these data suggests that loss of RASSF2 expression confers a more aggressive phenotype to lung cancer cells.

### 3.5. Loss of RASSF2 Expression Activates Growth Promoting Pathways

**Figure 5:**
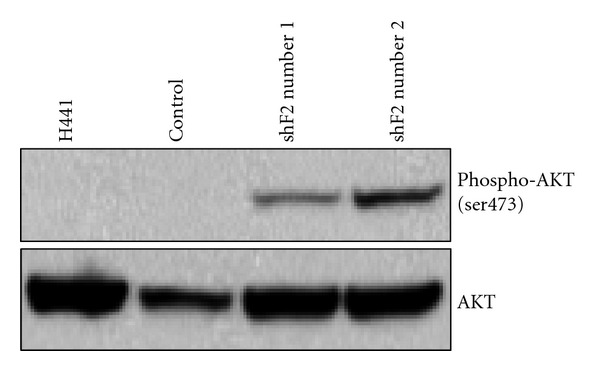
Loss of RASSF2 enhances Ras-mediated signaling pathways. Lysates from the control H441 cells and those stably transfected with the RASSF2 shRNA constructs were prepared, fractionated on SDS gels, and immunoblotted with antibodies against phosphorylated or total AKT. Loss of RASSF2 expression increased the levels of phosphorylated AKT.

Since loss of RASSF2 expression resulted in enhanced growth and transformation, we reasoned that inactivation of RASSF2 activated growth promoting pathways. In an effort to determine which prosurvival pathways were activated in the H441 cells knocked down for RASSF2, we analyzed the phosphorylation status of AKT in these cells. Western Blot analysis showed that levels of phosphorylated AKT increased in the cells stably expressing the RASSF2 shRNA constructs relative to control cells (Figure 5). Previous studies have found an association between the methylation status of RASSF2 and the levels of activated AKT. Oral squamous cell carcinomas in which *RASSF2* is methylated showed higher levels of activated AKT [18]. Taken together, our results and those from previous reports suggest that loss of RASSF2 expression results in activation of growth promoting pathways.

### 3.6. Loss of RASSF2 Expression Confers Resistance to Chemotherapeutic Agents

**Figure 6:**
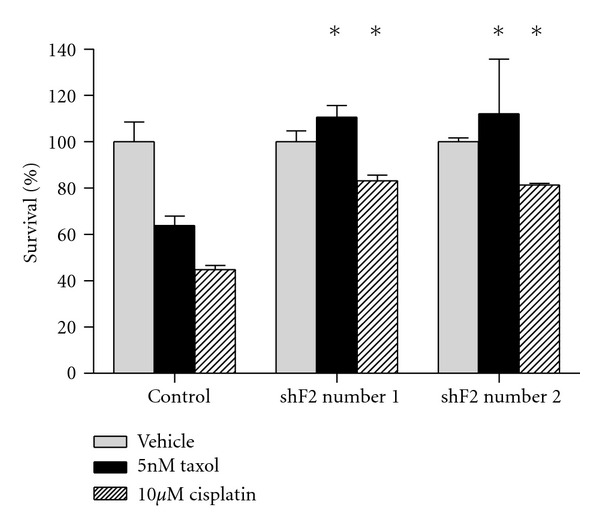
Inactivation of RASSF2 confers resistance to cisplatin and taxol. H441 cells stably transfected with control and RASSF2 shRNA constructs were seeded at 2 × 10^4^ cells per well in 12-well plates and treated with 5 nM taxol or 10 *μ*M cisplatin for 3 days. Cell death was estimated by trypan blue exclusion. Cells stably transfected with the RASSF2 shRNA showed significantly less cell death (*P* < 0.05) compared to the control cells for both taxol and cisplatin treatments.

To determine whether the more aggressive phenotype of the RASSF2 knockdown cells altered their response to chemotherapeutic agents, we treated the cells with taxol or cisplatin, two drugs commonly used in the treatment of nonsmall cell lung cancer, and measured their effects on cell death. Both taxol and cisplatin resulted in increased cell death in the control cells by approximately 40% and 50%, respectively. However, in the cells stably knocked down for RASSF2, taxol had no effect on cell growth and the cisplatin-induced cell death was somewhat abrogated (Figure 6). Thus, loss of RASSF2 expression confers resistance to taxol and cisplatin.

## 4. Discussion

RASSF2 is a novel K-Ras-specific effector that negatively regulates Ras signaling. It has the properties of a tumor suppressor with effects on apoptosis, cell cycle, and cell migration [9, 11, 23, 24]. It may play an important role in tumorigenesis as its expression is silenced in many tumor types by promoter methylation [4, 6, 8, 11–14, 17–19]. Moreover, inactivation of RASSF2 may be an early event in tumorigenesis as it is found inactivated in a high proportion of colon adenomas as well as early stages of prostate cancer [13, 17, 24], raising the possibility that loss of function of RASSF2 may be an initiating event in the development of certain tumor types.

To determine the effects of inactivation of RASSF2 on the transformed phenotype, we established a cell line in which we stably knocked down RASSF2 expression with RNAi technology. The cells in which RASSF2 had been inactivated adopted a more aggressive phenotype as evidenced by their enhanced growth in traditional 2-dimensional culture as well as their ability to grow in an anchorage independent manner. Consistent with this more transformed phenotype, the RASSF2 knockdown cells were less adherent than control cells, had an altered morphology, and showed an increased invasive potential. These results confirm and support previous studies showing that overexpression of RASSF2 inhibits growth, migration, and transformation [9, 11, 18, 23, 24].

The molecular mechanisms by which RASSF2 inhibits growth are not fully understood. Since RASSF2 has no inherent enzymatic activity or DNA binding properties, it more than likely acts by interacting with other proapoptotic effectors thereby modulating growth inhibitory pathways, much like the better characterized RASSF1A [1, 3]. We have previously shown that RASSF2 forms a direct and physiologically relevant complex with the proapoptotic effector PAR-4 [13], thereby modulating PAR-4 function. Other reports have shown that RASSF2 interacts with the Mst1/2 kinases, thereby regulating the Hippo signaling pathway [14, 15]. Thus, RASSF2 may act as a scaffold integrating multiple tumor suppressor pathways.

There is now conclusive evidence to support RASSF2 as a K-Ras-specific effector. RASSF2 binds to K-Ras in a GTP-dependent manner [11], and our data shows that RASSF2 and K-Ras form an endogenous complex (Figure 1). Furthermore, RASSF2 and K-Ras have been shown to interact at physiologically relevant levels in primary tissue [12]. Moreover, H441 lung cancer cells harbor a mutant K-Ras, and loss of RASSF2 expression in these cells dramatically enhanced their transformed phenotype. This data supports a previous study showing that inactivation of RASSF2 enhances K-Ras-mediated cell transformation in rat kidney cells [24]. It is now becoming clear that oncogenic K-Ras can both promote cellular proliferation as well as stimulate apoptosis [32]. Thus, RASSF2 may serve to keep the growth promoting activity of oncogenic K-Ras in check and loss of RASSF2 expression may then allow the growth promoting effects of activated K-Ras to dominate and override its growth suppressive effects.

In an effort to determine the mechanism behind the aggressive phenotype of the H441 cells in which RASSF2 levels were decreased, we examined the activation status of Ras-controlled signaling pathways and found an increase in activated AKT (Figure 5). This result is consistent with previous studies showing that cell lines in which the *RASSF2* promoter is methylated had higher levels of activated AKT compared to those cell lines in which the RASSF2 promoter was not methylated [18]. Interestingly, no effect on MAPK signaling molecules was observed in cells from *RASSF2* knockout mice during osteoblast differentiation [26]. Thus, it appears that the effects of RASSF2 in modulating Ras-mediated signaling pathways may be somewhat specific. Since RASSF2 can interact directly with activated K-Ras, it remains to be determined exactly how RASSF2 can selectively regulate some Ras-mediated signaling pathways while having little effect on others. RASSF2 interacts preferentially with K-Ras [11] and may thus negatively impact K-Ras-specific signaling pathways without impacting those pathways mediated by H-Ras or N-Ras. It is possible that RASSF2 may have some direct effects on the regulation of AKT activity, but further studies are required to determine whether this is indeed the case.

One possible explanation for the increased growth and transformed phenotype of the RASSF2 knockdown cells is enhanced NF-*κ*B signaling which may be promoted by inactivation of RASSF2. RASSF2 can modulate NF-*κ*B signaling by multiple mechanisms. Firstly, it forms a complex with I*κ*B*α* and *β* [26], thereby directly regulating the NF-*κ*B signaling pathway. Secondly, loss of RASSF2 is associated with elevated levels of activated AKT (Figure 5 and [18]), which can then activate NF-*κ*B signaling. AKT promotes tumor cell invasion which can occur via NF-*κ*B signaling [33–36]. Thirdly, inactivation of PAR-4 results in aberrant NF-*κ*B signaling [37], and we have shown that RASSF2 is required for the full apoptotic effects of PAR-4 [13]. Thus, RASSF2 may regulate NF-*κ*B signaling both directly and indirectly, and loss of RASSF2 expression results in deregulated NF-*κ*B signaling that may be associated with enhanced growth and invasion.

Our data also suggest that loss of RASSF2 expression confers resistance to taxol and cisplatin (Figure 6), 2 frontline therapeutics for the treatment of NSCLC [38]. These two agents offer only a modest improvement in median survival time for patients with advanced NSCLC [38]. Since RASSF2 is inactivated at a high frequency in lung cancer [9, 11, 19] and loss of RASSF2 expression is associated with an increase in activated AKT (Figure 5 and [18], a targeted therapeutic approach using agents against AKT, perhaps in combination with cytotoxic therapy, may prove more successful in at least a subset of lung cancer patients. Currently, there are a number of AKT inhibitors available, some of which are already in clinical trials [39].

In summary, we found that loss of RASSF2 expression enhances the transformed phenotype of lung cancer cells expressing oncogenic K-Ras. This more aggressive phenotype is associated with an increase in activated AKT, suggesting that RASSF2 can negatively regulate Ras-controlled growth promoting pathways. Inactivation of RASSF2 also confers resistance to cisplatin and taxol, suggesting that RASSF2, or the signaling pathways that it regulates, may serve as a target for therapy for lung cancer.

## References

[1] Donninger H, Vos MD, Clark GJ (2007). The RASSF1A tumor suppressor. *Journal of Cell Science*.

[2] Underhill-Day N, Hill V, Latif F (2011). N-terminal RASSF family (RASSF7-RASSF10): a mini review. *Epigenetics*.

[3] van der Weyden L, Adams DJ (2007). The Ras-association domain family (RASSF) members and their role in human tumourigenesis. *Biochimica et Biophysica Acta*.

[4] Falvella FS, Manenti G, Spinola M (2006). Identification of RASSF8 as a candidate lung tumor suppressor gene. *Oncogene*.

[5] Hesson LB, Dunwell TL, Cooper WN (2009). The novel RASSF6 and RASSF10 candidate tumour suppressor genes are frequently epigenetically inactivated in childhood leukaemias. *Molecular Cancer*.

[6] Hill VK, Underhill-Day N, Krex D (2011). Epigenetic inactivation of the RASSF10 candidate tumor suppressor gene is a frequent and an early event in gliomagenesis. *Oncogene*.

[7] Lock FE, Underhill-Day N, Dunwell T (2010). The RASSF8 candidate tumor suppressor inhibits cell growth and regulates the Wnt and NF-*κ*B signaling pathways. *Oncogene*.

[8] Schagdarsurengin U, Richter AM, Hornung J, Lange C, Steinmann K, Dammann RH (2010). Frequent epigenetic inactivation of RASSF2 in thyroid cancer and functional consequences. *Molecular Cancer*.

[9] Cooper WN, Dickinson RE, Dallol A (2008). Epigenetic regulation of the ras effector/tumour suppressor RASSF2 in breast and lung cancer. *Oncogene*.

[10] Kumari G, Singhal PK, Rao MRKS, Mahalingam S (2007). Nuclear transport of Ras-associated tumor suppressor proteins: different transport receptor binding specificities for arginine-rich nuclear targeting signals. *Journal of Molecular Biology*.

[11] Vos MD, Ellis CA, Elam C, Ulku AS, Taylor BJ, Clark GJ (2003). RASSF2 is a novel K-Ras-specific effector and potential tumor suppressor. *The Journal of Biological Chemistry*.

[12] Calvisi DF, Ladu S, Gorden A (2006). Ubiquitous activation of Ras and Jak/Stat pathways in human HCC. *Gastroenterology*.

[13] Donninger H, Hesson L, Vos M (2010). The ras effector RASSF2 controls the PAR-4 tumor suppressor. *Molecular and Cellular Biology*.

[14] Cooper WN, Hesson LB, Matallanas D (2009). RASSF2 associates with and stabilizes the proapoptotic kinase MST2. *Oncogene*.

[15] Song H, Oh S, Oh HJ, Lim DS (2010). Role of the tumor suppressor RASSF2 in regulation of MST1 kinase activity. *Biochemical and Biophysical Research Communications*.

[16] Endoh M, Tamura G, Honda T (2005). RASSF2, a potential tumour suppressor, is silenced by CpG island hypermethylation in gastric cancer. *British Journal of Cancer*.

[17] Hesson LB, Wilson R, Morton D (2005). CpG island promoter hypermethylation of a novel Ras-effector gene RASSF2A is an early event in colon carcinogenesis and correlates inversely with K-ras mutations. *Oncogene*.

[18] Imai T, Toyota M, Suzuki H (2008). Epigenetic inactivation of RASSF2 in oral squamous cell carcinoma. *Cancer Science*.

[19] Kaira K, Sunaga N, Tomizawa Y (2007). Epigenetic inactivation of the RAS-effector gene RASSF2 in lung cancers. *International Journal of Oncology*.

[20] Maruyama R, Akino K, Toyota M (2008). Cytoplasmic RASSF2A is a proapoptotic mediator whose expression is epigenetically silenced in gastric cancer. *Carcinogenesis*.

[21] Park HW, Hio CK, Kim IJ (2007). Correlation between hypermethylation of the RASSF2A promoter and K-ras/BRAF mutations in microsatellite-stable colorectal cancers. *International Journal of Cancer*.

[22] Steinmann K, Sandner A, Schagdarsurengin U, Dammann RH, Dammann RH (2009). Frequent promoter hypermethylation of tumor-related genes in head and neck squamous cell carcinoma. *Oncology Reports*.

[23] Zhang Z, Sun D, van Do N, Tang A, Hu L, Huang G (2007). Inactivation of RASSF2A by promoter methylation correlates with lymph node metastasis in nasopharyngeal carcinoma. *International Journal of Cancer*.

[24] Akino K, Toyota M, Suzuki H (2005). The ras effector RASSF2 is a novel tumor-suppressor gene in human colorectal cancer. *Gastroenterology*.

[25] Yi Y, Nandana S, Case T (2009). Candidate metastasis suppressor genes uncovered by array comparative genomic hybridization in a mouse allograft model of prostate cancer. *Molecular Cytogenetics*.

[26] Song H, Kim H, Lee K (2012). blation of Rassf2 induces bone defects and subsequent haematopoietic anomalies in mice. *The EMBO Journal*.

[27] Tommasi S, Dammann R, Zhang Z (2005). Tumor susceptibility of Rassf1a knockout mice. *Cancer Research*.

[28] van der Weyden L, Tachibana KK, Gonzalez MA (2005). The RASSF1A isoform of RASSF1 promotes microtubule stability and suppresses tumorigenesis. *Molecular and Cellular Biology*.

[29] Rodenhuis S, van de Wetering ML, Mooi WJ (1987). Mutational activation of the K-ras oncogene. A possible pathogenetic factor in adenocarcinoma of the lung. *The New England Journal of Medicine*.

[30] Nosho K, Yamamoto H, Takahashi T (2007). Genetic and epigenetic profiling in early colorectal tumors and prediction of invasive potential in pT1 (early invasive) colorectal cancers. *Carcinogenesis*.

[31] Humphries MJ (2001). Cell adhesion assays. *Applied Biochemistry and Biotechnology Part B*.

[32] Cox AD, Der CJ (2003). The dark side of Ras: regulation of apoptosis. *Oncogene*.

[33] Julien S, Puig I, Caretti E (2007). Activation of NF-*κ*B by Akt upregulates snail expression and induces epithelium mesenchyme transition. *Oncogene*.

[34] Kim D, Kim S, Koh H (2001). Akt/PKB promotes cancer cell invasion via increased motility and metalloproteinase production. *FASEB Journal*.

[35] Park BK, Zeng X, Glazer RI (2001). Akt1 induces extracellular matrix invasion and matrix metalloproteinase-2 activity in mouse mammary epithelial cells. *Cancer Research*.

[36] Romashkova JA, Makarov SS (1999). NF-*κ*B is a target of AKT in anti-apoptotic PDGF signalling. *Nature*.

[37] Garcia-Cao I, Lafuente MJ, Criado LM, Diaz-Meco MT, Serrano M, Moscat J (2003). Genetic inactivation of Par4 results in hyperactivation of NF-*κ*B and impairment of JNK and p38. *The EMBO Reports*.

[38] Schiller JH, Harrington D, Belani CP (2002). Comparison of four chemotherapy regimens for advanced non-small-cell lung cancer. *The New England Journal of Medicine*.

[39] Hirai H, Sootome H, Nakatsuru Y (2010). MK-2206, an allosteric akt inhibitor, enhances antitumor efficacy by standard chemotherapeutic agents or molecular targeted drugs *in vitro* and *in vivo*. *Molecular Cancer Therapeutics*.

